# Architectural Grade Combined With Spread Through Air Spaces (STAS) Predicts Recurrence and is Suitable for Stratifying Patients Who Might Be Eligible for Lung Sparing Surgery for Stage I Adenocarcinomas

**DOI:** 10.1007/s12253-020-00855-7

**Published:** 2020-06-20

**Authors:** Tamás Zombori, Anita Sejben, László Tiszlavicz, Gábor Cserni, Regina Pálföldi, Edit Csada, József Furák

**Affiliations:** 1 Department of Pathology, Faculty of Medicine, University of Szeged, Állomás u. 1., H6725 Szeged, Hungary; 2 Department of Pathology, Bács-Kiskun County Teaching Hospital, Nyíri út 38, Kecskemét, H6000 Hungary; 3 Csongrád County Hospital of Chest Diseases, Alkotmány u. 36. , Deszk, H6772 Hungary; 4 Department of Surgery, University of Szeged, Semmelweis u. 8., Szeged, H6720 Hungary

**Keywords:** Lung adenocarcinoma, Spread through airspaces (STAS), Architectural grade, Lung sparing surgery, Sublobar resection

## Abstract

The spread through air spaces (STAS) has a main role in local recurrence of stage I lung adenocarcinomas (LAs), therefore its presence might question sublobar resection as a therapeutic option. The aim of our study was to evaluate the distribution of STAS in stage I LAs, to stratify patients according to local recurrence and to identify a group of patients who might be suitable for sublobar surgery. Patients resected with LA were included. The presence of STAS was recorded on hematoxylin eosin stained slides and clinicopathological data were obtained from medical charts. Overall survival (OS) and disease-free survival (DFS) were registered. Statistical methods included Kruskal-Wallis tests, Kaplan-Meier analyses, log-rank tests and Cox-regressions. 292 patients were included. STAS was identified in 38.7% and 95.7% of micropapillary carcinomas showed STAS. Significant correlation was found between STAS and high-grade patterns. Significant differences were found between OS and DFS estimates of STAS0 and STAS1 cases (5-y-OS: 80.0% vs. 68.4%; 5-y-DFS: 71.1% vs. 57.1%). The presence of STAS was associated with unfavorable prognosis in low and intermediate architectural grades, but not in high-grade. Multivariate analysis revealed that architectural grade (HR_(OS)_:2.09; HR_(DFS)_:1.52) and STAS (HR_(OS)_:1.51; HR_(DFS)_:1.48) were independent prognostic markers in stage I LA. Architectural grade combined with STAS was superior to other prognostic grades. The combination of architectural grade and STAS proved to be a prognostic factor that is superior to previously introduced grading systems. Patients having low and intermediate grade LAs without STAS might be eligible for sublobar resection.

**Electronic supplementary material:**

The online version of this article (10.1007/s12253-020-00855-7) contains supplementary material, which is available to authorized users.

## Background

Despite the development of molecular targeted therapies and immune checkpoint inhibitors for the treatment of pulmonary adenocarcinoma, its outcome is still unfavorable [1]. Lobar resection with lymph node dissection remains the most common curative therapy in stage I disease [2, 3]. There are several studies in progress aiming to validate the utility of lung sparing or sublobar resection for early stage lung adenocarcinoma and to answer whether lung sparing resection for this disease is only a function preserving or a curative treatment option as well [4]. These ongoing Japanese trials have suggested, that sublobar resection achieves local control and recurrence-free survival in patients with radiologically noninvasive lung cancer, with a maximum tumor diameter of ≤ 2 cm and a solid tumor ratio of ≤ 0.25 defined with CT [4, 5].

Spread through air spaces (STAS) is a recently described pattern of invasion of lung neoplasms [6]. STAS represents micropapillary clusters, solid nests or single cells beyond the edge of the tumor invading into air spaces [7, 8]. STAS was implemented in the 2015 World Health Organization Classification of Lung Tumors, and in the 8th edition of the Cancer Staging Manual of the American Joint Committee on Cancer (AJCC), resulting in the refining of the definition of tumor invasion and furthermore the criteria of in situ, minimally invasive and invasive adenocarcinoma, as well [9, 10]. Although STAS is extensively studied nowadays, the pathomechanism is yet unknown. Cellular dedifferentiation, loss of cell membrane cohesion and mechanical impact by the surgeon have been proposed in the etiology of STAS [11]. Though the development of STAS is debated, an unfavorable prognostic impact on survival was demonstrated in lung adenocarcinomas with STAS by several reports [7, 12–16]. STAS has been associated with adverse prognosis in early stage lung adenocarcinomas in patients, who underwent sublobar resections [7, 17]. In contrast to these results, Uruga and coworkers have not found such an impact in cases of sublobar surgery.

Even though, lobectomy is the standard therapy for lung adenocarcinoma in early stage, the process itself might be high-risk for patients with comorbidities, namely chronic obstructive lung diseases, bronchiectasis or severe cardiovascular diseases. Sublobectomy may be a treatment option for these patients, in selected cases of lung adenocarcinoma lacking STAS. The aim of our study was to evaluate the distribution of STAS among different subtypes of stage I lung adenocarcinoma; to analyze the impact of morphologic features and prognostic systems on survival; to stratify patients according to local recurrence and to identify a group of patients who are suitable for lung sparing surgery.

## Methods

Patients operated on at the Department of Surgery, University of Szeged between 2004 and 2013 with stage I lung adenocarcinoma were included in our retrospective, consecutive series. Exclusion criteria were multicentric, metachronous or metastatic tumors, variants of adenocarcinoma (namely invasive mucinous, mixed invasive mucinous/non-mucinous, colloid, fetal, enteric adenocarcinoma and sarcomatoid carcinoma), patients who underwent lung cancer surgery in the previous 2 years, positive surgical margins, perioperative death, metastasis to lymph nodes and vascular invasion.

**Fig. 1:**
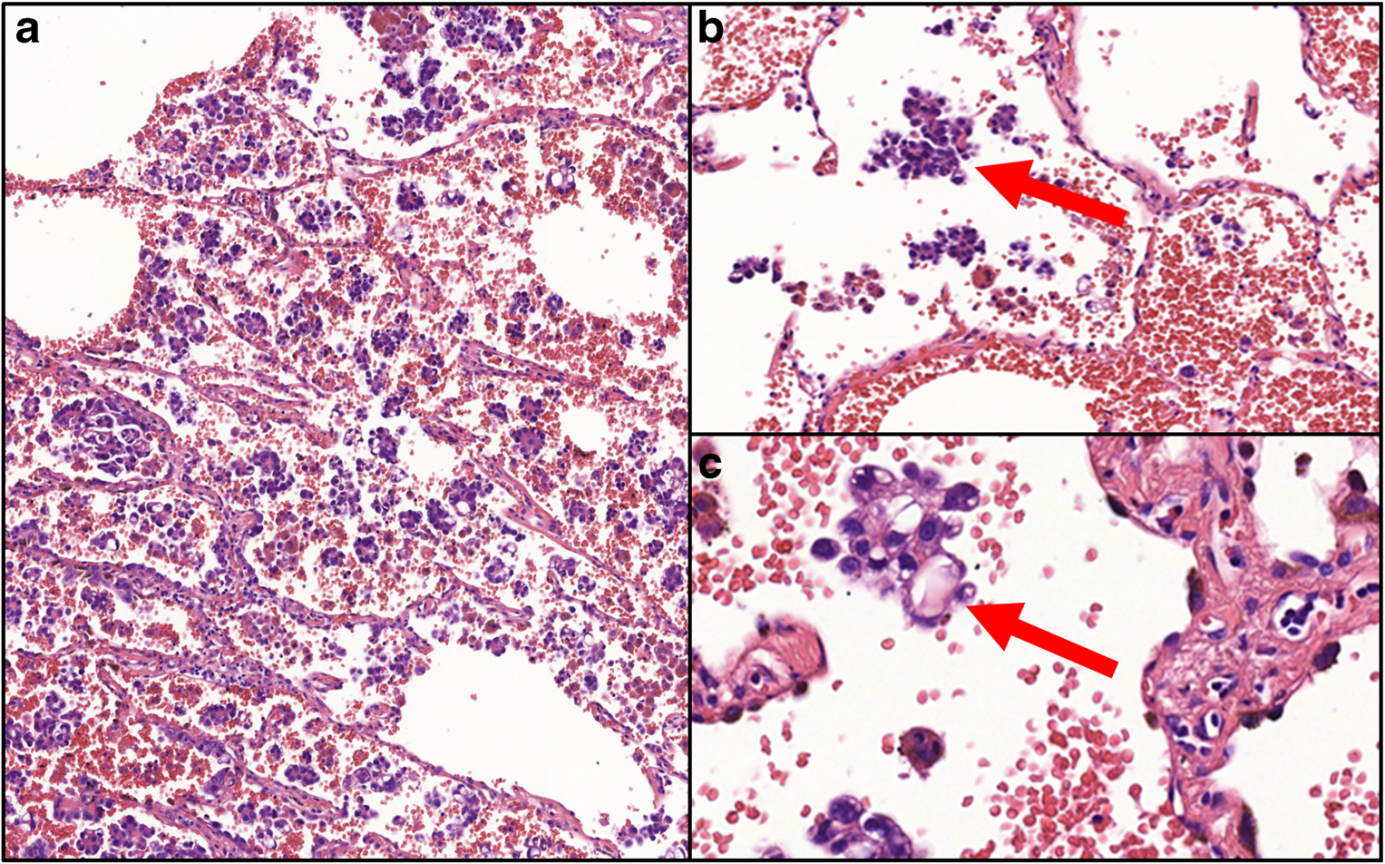
**a** Micropapillary adenocarcinoma at small magnification (HE, 10x); **b** Presence of intraalveolar atypical tumor cells (arrow) beyond the border of the tumor (HE, 20x); **c** Cluster of tumor cells (arrow) at high magnification (HE, 40x)

All hematoxylin eosin slides of the patients included were evaluated and revised if needed, according to the WHO Classification of Lung Tumors [9]. The presence of STAS was recorded by two pathologists (TZ, LT), who were blinded to patients’ clinical outcome and discrepancies were discussed at a multi-headed microscope (Olympus BX43, Tokyo, Japan). STAS was defined as micropapillary structures or solid nests that were present in the air spaces beyond the edge of the tumor, even if in the first alveoli from tumor edge [7]. Figure 1 presents a micropapillary adenocarcinoma with STAS. The intraalveolar tumor clusters found distant from the tumor without alveolar interconnection to the tumor mass, random clusters of tumor cells especially at the edges of the sample, jagged edges of tumor clusters, linear strips of cells lifted off alveolar walls were identified as ex vivo artifacts [7, 11].

Clinical data, including gender, age, tumor localization, type of surgery, smoking habits, site of recurrence and follow-up data were obtained from medical charts, meanwhile additional histopathological features including presence of nuclear atypia, lymphovascular invasion and necrosis, and prognostic systems, namely architectural grade, Kadota-grade and Sica-grade were collected from histopathology reports. Stage I was defined according to the 8th edition of the Cancer Staging Manual by the AJCC [10], namely tumors with diameter ≤ 4 cm, without any metastasis (pT1a-b-c-pT2a-pN0-pM0). Lymphadenectomy was part of the operation in all cases and lymph nodes were examined histologically.

The follow-up of patients consisted of three-monthly physical examination, chest X-ray and abdominal ultrasonography evaluation in the first 2 years, then every 6 months until the fifth year. Chest CT was performed every 6 months for the first 2 years, then 6 or 12 monthly, depending on the patient, until the fifth year. In case of any suspicion of progression, chest CT and abdominal ultrasonography were included. The follow-up period ended on 31st August 2019.

Concerning the statistical correlation of STAS and subtypes of adenocarcinoma, the Kruskal-Wallis test was applied. The impact of STAS on DFS and OS was assessed by the Kaplan-Meier method. The log-rank test was used for pairwise comparisons. Univariate and multivariate Cox regressions were utilized for analyzing the clinicopathological variables. All statistical tests were two-sided, and p < 0.05 values were considered statistically significant. The SPSS Statistics software (IBM, SSPS 23.0, Armonk, NY USA) was used for the analyses.

This retrospective study was approved by the institutional ethical committee of the Albert Szent-Györgyi Clinical Centre of the University of Szeged (ethical approval: 58/2020-SZTE).

## Results

Altogether 292 patients matched the inclusion criteria. The average age of patients with stage I lung adenocarcinoma was 62.7 years (median: 62.3 years, range: 32–86). Relevant clinical data are presented in Table 1. No gender predilection was found in our series. Most patients were treated with lobectomy and most were actual or previous heavy smokers for decades. The most frequent subtype was solid adenocarcinoma, followed by lepidic adenocarcinoma. Lymphovascular spread was identified in nearly every fourth case and more than half of the cases were diagnosed with stage IA disease. The median follow-up was 75.7 months (range: 12–187 months). Tumor specific death was observed in 81 patients, while recurrence was detected in 110 cases. In the latter group, clinical information was available in 72 cases about the site of recurrence. Recurrence in hilar or mediastinal lymph nodes, in lungs and disseminated disease were found in 10 (13.9%), 21 (29.2%) and in 41 (56.9%) cases, respectively. The mean and median of OS and DFS were 58.6 months, 52.4 months and 57.1 months 54.3 months, respectively.

**Table 1:** Clinicopathological characteristics of patients evaluated and results of univariate Cox regression regarding the different variables

Clinicopathological data	n	%	OS			DFS		
			p	HR	95%CI	p	HR	95%CI
Gender			0.22	0.76	0.48–1.17	0.41	0.85	0.58–1.24
Female	154	52.7						
Male	138	47.3						
Localization			0.68	1.09	0.69–1.72	0.2	1.2	0.87–1.86
Left	181	62						
Right	111	38						
Type of surgery			**< 0.001**	**3.53**	**1.97–6.36**	**< 0.001**	**2.98**	**1.74–5.11**
Lobar resection	266	91.1						
Sublobar resection	26	8.9						
Smoking habits			**0.006**	**2.84**	**1.35–5.96**	**0.001**	**2.97**	**1.56–5.60**
Heavy smoker	132	45.2						
Never	38	13						
No data	122	41.8						
Subtype			**< 0.001**	**1.59**	**1.32–1.92**	**< 0.001**	**1.35**	**1.16–1.58**
Solid	108	37						
Micropapillary	23	7.9						
Acinar	53	18.1						
Papillary	44	15						
Lepidic	64	22						
Necrosis			0.17	1.35	0.87–2.10	1.24	1.34	0.92–1.95
presence	133	45.5						
absence	159	54.5						
Lymphatic invasion			**0.05**	**1.61**	**1.00-2.60**	0.27	1.27	0.82–1.95
presence	68	23.3						
absence	224	76.7						
STAS			**0.04**	**1.53**	**1.10–2.43**	**0.04**	**1.47**	**1.01–2.13**
presence	123	42.1						
absence	169	57.9						
pT			**< 0.001**	**1.55**	**1.24–1.85**	**< 0.001**	**1.25**	**1.11–1.45**
T1a	18	6.1						
T1b	77	26.3						
T1c	75	25.7						
T2a	122	41.9						
Stage			**< 0.001**	**1.5**	**1.20–1.81**	**< 0.001**	**1.18**	**1.02–1.43**
IA1-3	182	62.3						
IB	110	37.7						

OS: overall survival, DFS: disease-free survival, HR: hazard ratio, 95%CI: 95% confidence interval, STAS: spread through air spaces

Table 2 displays the distribution of STAS among lung adenocarcinomas. STAS was identified in more than one third of all cases (n = 113; 38.7%); though it was present in all subtypes, STAS was found in more than 95% of micropapillary carcinomas.

**Table 2:** Distribution of STAS according to predominant growth patterns of lung adenocarcinoma

Predominant growth pattern	STAS1		STAS0	
	n	%	n	%
Solid	32	29.6	76	70.4
Micropapillary	22	95.7	1	4.3
Acinar	20	37.7	33	62.3
Papillary	16	36.4	28	63.6
Lepidic	23	35.9	41	64.1

STAS: spread through air spaces; STAS1: STAS present; STAS0: STAS absent

Pulmonary adenocarcinomas have more than one growth pattern in 80% of the cases. All growth pattern components were recorded in all cases. In tumors with STAS, the presence of micropapillary pattern was frequent (85.3%), followed by solid pattern as the second most common pattern (37.4%). Tumors with STAS were associated with high-grade (micropapillary or solid) growth pattern components in 91.7% of the cases. The Kruskal-Wallis test revealed significant correlation between STAS and high-grade growth patterns (p < 0.001).

In univariate Cox regression, subtype, presence of nuclear atypia, lymphovascular spread and STAS were found significant prognosticators (Table 1). Regarding the findings of the log rank test, significant differences were observed between OS and DFS estimates of cases with STAS and without STAS (5-y-OS: 80.0% vs. 68.4%; p = 0.045; 5-y-DFS: 71.1% vs. 57.1% p = 0.043), respectively. The prognostic impact of STAS was evaluated in each architectural grade, as well (Table 3). The presence of STAS was associated with unfavorable prognosis in low (p_(OS)_ = 0.04; p_(DFS)_ = 0.034) and intermediate architectural grade (p_(DFS)_ = 0.04), but not in high-grade. Multivariate analysis revealed that architectural grade (p_(OS)_ < 0.001; HR:2.09 95%CI:1.41–2.84; p_(DFS)_ = 0.003; HR:1.52 95%CI:1.15–2.01) and STAS (p_(OS)_ = 0.041; HR:1.51 95%CI:1.12–2.31; p_(DFS)_ = 0.045; HR:1.48 95%CI:1.19–2.03) were independent prognostic factors in stage I lung adenocarcinoma.

**Table 3:** The overall and disease-free survival estimates and the results of univariate Cox regression, regarding the different architectural grades with or without STAS

	n	OS (%)	p_OS_	HR_OS_	95%CI	DFS (%)	p_DFS_	HR_DFS_	95%CI
G1									
STAS0	37	96.7	**0.04**	**4.73**	**1.01–22.5**	93.9	**0.034**	**3.21**	**1.1–9.42**
STAS1	27	80				68.8			
G2									
STAS0	58	89.4	0.12	2.23	0.80–6.17	78.9	**0.04**	**2.03**	**1.1–4.2**
STAS1	40	76.6				56.9			
G3									
STAS0	74	64.7	0.58	1.16	0.68–1.98	57.4	0.92	1.02	0.62–1.68
STAS1	56	56.8				51.7			

G1: lepidic adenocarcinoma, G2: acinar and papillary adenocarcinoma, G3: solid and micropapillary adenocarcinoma, STAS: spread through air spaces, OS: overall survival, DFS: disease-free survival

**Fig. 2:**
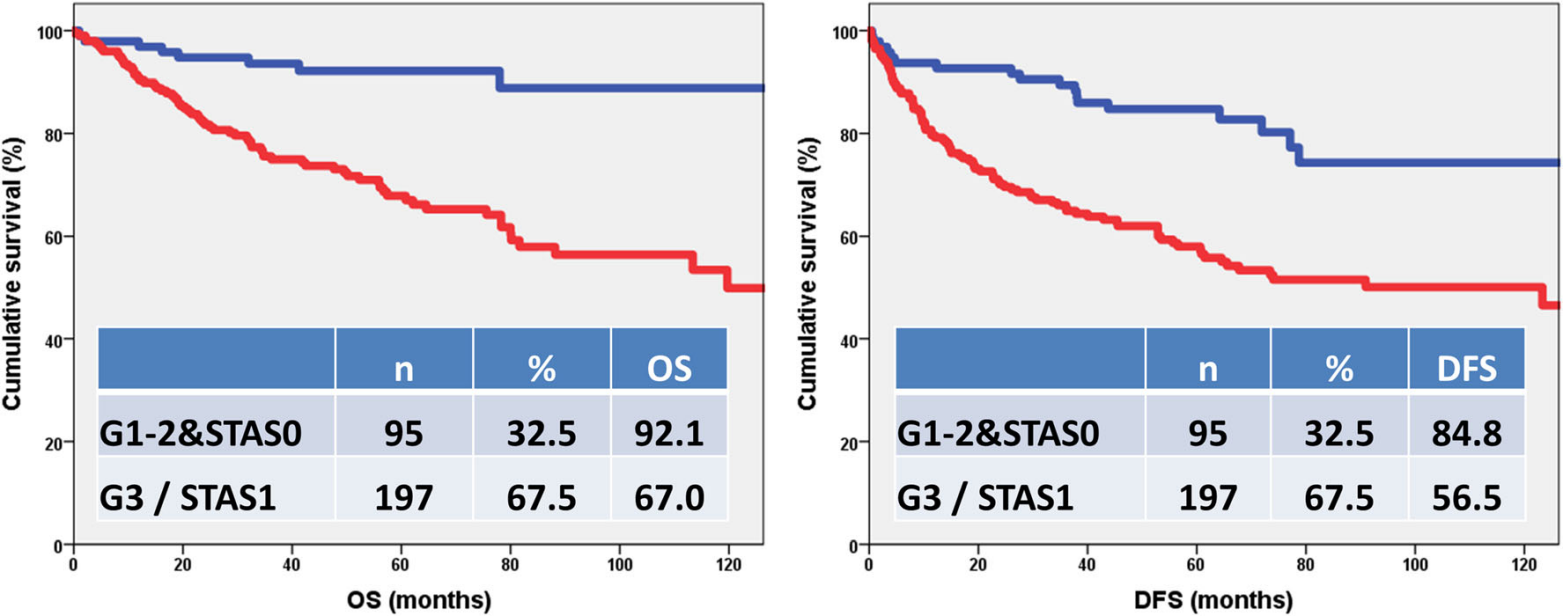
Kaplan-Meier test of architectural grade combined with STAS. The low- and intermediate-grade lung adenocarcinomas without STAS [G1-2&STAS0] have more favorable outcome than high-grade adenocarcinomas and/or adenocarcinomas with STAS [G3 / STAS1] (p_OS_<0.001, p_DFS_<0.001)

Figure 2 demonstrates that adenocarcinomas having high-grade morphology and/or STAS are associated with unfavorable prognosis, while tumors lacking these features have a more favorable outcome (p_(OS)_ < 0.001, p_(DFS)_ < 0.001). Identification of high-risk patients (grade 3 and/or STAS1) and those with low-risk for recurrence and tumor-related death (grade 1–2&STAS0) can become a powerful prognosticator.

In a previous study, the available grading systems were compared and correlated with survival data, and the architectural grade was found the most optimal grading system describing prognosis [18]. The multivariate analysis of architectural grade, Kadota-grade, Sica-grade and architectural grade combined with STAS has shown (Supplementary table 1), that the architectural grade combined with STAS is superior to the other prognostic systems (p_(OS)_ = 0.012; HR:2.98 95%CI:1.31–6.92; p_(DFS)_ = 0.009; HR:2.29 95%CI:1.21–4.04).

## Discussion

In keeping with previous studies, STAS was diagnosed in more than one third of all cases in our series [2, 7, 12, 13, 19]. The phenomenon was first described by Onozato and coworkers [20]. Using three dimensional mapping of the resected tumor, it was demonstrated that the areas of dissociated tumor cells are connected to each other and the main tumor mass, as well. Kadota et al. have proposed that this intraalveolar dissemination of tumor cells, namely STAS is a form of invasion [7]. Several studies have been published recently aiming at the validation of the prognostic impact of STAS. Warth and coworkers have found that STAS is a prognostic feature in all stages of lung adenocarcinoma [8].

Although the prognostic importance of STAS has been demonstrated, its development is still debated. The process of dedifferentiation may alter the expression of genes in cancer cells, resulting in divergent features compared to the initial cells. The adhesion between tumor cells may disrupt due to the process of dedifferentiation, resulting in discohesive cell clusters floating in the alveoli. These free-floating tumor cell clusters may deposit on the alveolar wall and induce stromal reaction and nidus formation. This theory supports the bad prognostic impact of STAS [11]. In contrast, some authors have an alternative explanation, namely that STAS is an artifact [21, 22]. Thunnissen and coworkers have introduced the concept of the spread through a knife surface (STAKS). They have demonstrated that artificial displacement during a knife cut is a real phenomenon, resulting in an increase of the number of intraalveolar tumor cell clusters with each sequential cut [21]. In keeping with the results of Thunnissen, Blaauwgeers et al. have found that less intraalveolar cell clusters are present in cases of fewer sections, meanwhile more floating cells were detected in specimens of sequential sectioning with the same prosecting blade [22]. Despite the theory of STAKS, our findings are in correspondence with others [12–16], namely STAS is an independent prognostic factor in stage I lung adenocarcinoma.

Lung adenocarcinomas with STAS are present mostly in male patients and are associated with high- grade histology, higher stage, lymphovascular spread and lymph node metastasis. STAS has been related with more frequent locoregional recurrence in sublobar resections [23, 24]. Regarding targetable molecular alterations, adenocarcinomas with STAS have been related to wild type epidermal growth factor receptor (EGFR) [12, 25, 26], ALK rearrangement [23, 26] and Kirsten rat sarcoma viral oncogene homolog (KRAS) mutation [20].

In consistency with others’ results [27–31], high architectural grade (solid and micropapillary tumors) was associated with an unfavorable outcome in our study. Furthermore, even a subdominant component of high-grade pattern is related to higher recurrence rate and tumor-related death [32].

We have demonstrated that patients having stage I lung adenocarcinomas with high architectural grade (solid and micropapillary carcinomas) and adenocarcinomas with STAS are at high-risk of recurrence and tumor-specific death, while patients having low- and intermediate-grade tumors without STAS have a more favorable outcome (p_(OS)_ < 0.001, p_(DFS)_ < 0.001). Furthermore, the combination of architectural grade with STAS was proven to be a prognostic system, superior to the previously tested architectural grade, Kadota-grade and Sica-grade. Patients having stage I, low- or intermediate-grade lung adenocarcinoma without STAS might be suitable for lung sparing surgery.

Being a histopathological feature, the preoperative detection of STAS is controversial. Core needle biopsy specimen might be able to reveal STAS preoperatively, however, there is a high chance of sampling error with this method. Unfortunately, bronchoalveolar lavage does not extract significant number of tumor cells for cytological examinations [33]. Another option is the evaluation of STAS and high-grade morphology on intraoperative frozen sections. Yeh and coworkers have evaluated the sensitivity and specificity of the growth pattern recognition on intraoperative frozen sections. They reported a sensitivity of 69% for solid, and 37% for micropapillary patterns, with a specificity of 96% and 97%, respectively [34]. Eguchi et al. observed the demonstrability of STAS on intraoperative frozen sections with a sensitivity of 71% and a specificity of 92% for detecting STAS [24].

Prospective studies are required for the evaluation of sensitivity and specificity of detecting tumors with high-grade morphology and/or STAS on frozen sections. Multiple frozen sections are needed due to the fact, that two features have to be defined, namely the growth pattern of the tumor and the presence of STAS in the surrounding lung parenchyma. Morimoto and coworkers have demonstrated, that STAS around the tumor is present in an area that is smaller than the diameter of the tumor, furthermore STAS was not present in the area of lung parenchyma farther than 7.3 mm from the edges of the tumor [35]. Therefore, at least two frozen sections are required, one from the tumor mass and one from its neighboring lung parenchyma.

In selected patients having stage I lung adenocarcinoma, lung sparing surgery might be a treatment option, if high architectural grade and STAS are not detected in the preoperative biopsy and on the intraoperative frozen section. If either high-grade morphology or STAS is present in the preoperative biopsy, primary lobectomy should be performed. If these components are identified during intraoperative examination, completing lobectomy can be suggested in one session.

The retrospective nature of the series, being a single-institutional study, the lack of evaluation of surgical margins and molecular alterations, the low number of sublobar resections and the exclusion of cases with vascular invasion may be limitations. The cases with positive resections margins are rare in our center in stage I adenocarcinomas, therefore these cases were excluded. The low rate of sublobar resections is due to international and national guidelines [36, 37]. The tumors demonstrating vascular invasion have an unfavorable prognosis even in small tumors [38], therefore these cases were excluded as well.

Our study has also strengths: it is based on a relatively large cohort; tumors were sliced with prosecting blades cleaned before each slice, aiming at the minimization of the STAKS effect; and the definition of STAS and the exclusion of artifacts were rigorously applied.

In our retrospective study of consecutive stage I adenocarcinomas, STAS was proven as a significant prognostic factor in univariate analysis. Architectural grade and STAS were independent prognostic factors in multivariate analysis, as well. The combination of architectural grade with STAS resulted in a prognostic system superior to previously introduced grading systems. Patients having low- and intermediate-grade lung adenocarcinomas without STAS might be candidates for lung sparing surgery.

## Electronic Supplementary Material

ESM 1 (DOCX 12 kb)

## Data Availability

The datasets generated during and/or analysed during the current study are available from the corresponding author on reasonable request.

## References

[1] Siegel RL, Miller KD, Jemal A (2019). Cancer statistics, 2019. CA Cancer J Clin.

[2] Toyokawa G, Yamada Y, Tagawa T, Oda Y (2018). Significance of spread through air spaces in early-stage lung adenocarcinomas undergoing limited resection. Thorac Cancer.

[3] Ginsberg RJ, Rubinstein LV (1995). Randomized trial of lobectomy versus limited resection for T1 N0 non-small cell lung cancer. Lung Cancer Study Group. Ann Thorac Surg.

[4] Aokage K, Yoshida J, Hishida T, Tsuboi M, Saji H, Okada M, Suzuki K, Watanabe S, Asamura H (2017). Limited resection for early-stage non-small cell lung cancer as function-preserving radical surgery: a review. Jpn J Clin Oncol.

[5] Suzuki K, Watanabe S, Wakabayashi M, Moriya Y, Yoshino I, Tsuboi M, Mitsudomi T, Asamura H (2017) A nonrandomized confirmatory phase III study of sublobar surgical resection for peripheral ground glass opacity dominant lung cancer defined with thoracic thin-section computed tomography (JCOG0804/WJOG4507L). J Clin Oncol 35: Abstract 8561

[6] Travis WD, Brambilla E, Nicholson AG, Yatabe Y, Austin JHM, Beasley MB, Chirieac LR, Dacic S, Duhig E, Flieder DB, Geisinger K, Hirsch FR, Ishikawa Y, Kerr KM, Noguchi M, Pelosi G, Powell CA, Tsao MS, Wistuba I, WHO Panel (2015). The 2015 World Health Organization classification of lung tumors: Impact of genetic, clinical and radiologic advances since the 2004 Classification. J Thorac Oncol.

[7] Kadota K, Nitadori J, Sima CS, Ujiie H, Rizk NP, Jones DR, Adusumilli PS, Travis WD (2015). Tumor spread through air spaces is an important pattern of invasion and impacts the frequency and location of recurrences after limited resection for small stage I lung adenocarcinomas. J Thorac Oncol.

[8] Warth A (2017). Spread through air spaces (STAS): a comprehensive update. Transl Lung Cancer Res.

[9] Travis WD, Brambilla E, Burke AP, Marx A, Nicholson AG (2015) WHO classification of tumours of the lung, pleura, thymus and heart. Fourth edition. International Agency for Research on Cancer, Lyon 10.1097/JTO.0000000000000663 26291007

[10] Amin MB, Edge S, Greene F, Byrd DR, Brookland RK, Washington MK, Gershenwald JE, Compton CC, Hess KR, Sullivan DC, Jessup JM, Brierley JD, Gaspar LE, Schilsky RL, Balch CM, Winchester DP, Asare EA, Madera M, Gress DM, Meyer LR (2018) AJCC Cancer Staging Manual. Vol xvii. 8th ed. Springer International Publishing, Cham

[11] Shih AR, Mino-Kenudson M (2020). Updates on spread through air spaces (STAS) in lung cancer. Histopathology.

[12] Shiono S, Yanagawa N (2016). Spread through air spaces is a predictive factor of recurrence and a prognostic factor in stage I lung adenocarcinoma. Interact Cardiovasc Thorac Surg.

[13] Uruga H, Fujii T, Fujimori S, Kohno T, Kishi K (2017). Semiquantitative assessment of tumor spread through air spaces (STAS) in early-stage lung adenocarcinomas. J Thorac Oncol.

[14] Kadota K, Kushida Y, Katsuki N, Ishikawa R, Ibuki E, Motoyama M, Nii K, Yokomise H, Bandoh S, Haba R (2017). Tumor spread through air spaces is an independent predictor of recurrence-free survival in patients with resected lung squamous cell carcinoma. Am J Surg Pathol.

[15] Lu S, Tan KS, Kadota K, Eguchi T, Bains S, Rekhtman N, Adusumilli PS, Travis WD (2017). Spread through air spaces (STAS) is an independent predictor of recurrence and lung cancer-specific death in squamous cell carcinoma. J Thorac Oncol.

[16] Morimoto J, Nakajima T, Suzuki H, Nagato K, Iwata T, Yoshida S, Fukuyo M, Ota S, Nakatani Y, Yoshino I (2016). Impact of free tumor clusters on prognosis after resection of pulmonary adenocarcinoma. J Thorac Cardiovasc Surg.

[17] Shiono S, Endo M, Suzuki K, Yarimizu K, Hayasaka K, Yanagawa N (2018). Spread through air spaces is a prognostic factor in sublobar resection of non-small cell lung Cancer. Ann Thorac Surg.

[18] Zombori T, Furák J, Nyári T, Cserni G, Tiszlavicz L (2018). Evaluation of grading systems in stage I lung adenocarcinomas: a retrospective cohort study. J Clin Pathol.

[19] Dai C, Xie H, Su H, She Y, Zhu E, Fan Z, Zhou F, Ren Y, Xie D, Zheng H, Kadeer X, Chen D, Zhang L, Jiang G, Wu C, Chen C (2017). Tumor spread through air spaces affects the recurrence and overall survival in patients with lung adenocarcinoma > 2 to 3 cm. J Thorac Oncol.

[20] Onozato ML, Kovach AE, Yeap BY, Morales-Oyarvide V, Klepeis VE, Tammireddy S, Heist RS, Mark EJ, Dias-Santagata D, Iafrate AJ, Yagi Y, Mino-Kenudson M (2013). Tumor islands in resected early-stage lung adenocarcinomas are associated with unique clinicopathologic and molecular characteristics and worse prognosis. Am J Surg Pathol.

[21] Thunnissen E, Blaauwgeers HJ, de Cuba EM, Yick CY, Flieder DB (2016). Ex vivo artifacts and histopathologic pitfalls in the lung. Arch Pathol Lab Med.

[22] Blaauwgeers H, Flieder D, Warth A, Harms A, Monkhorst K, Witte B, Thunnissen E (2017) A prospective study of loose tissue fragments in non-small cell lung cancer resection specimens: An alternative view to “spread through air spaces”. Am J Surg Pathol 41:1226–1230. 10.1097/PAS.0000000000000889 10.1097/PAS.0000000000000889 28622180

[23] Kadota K, Kushida Y, Kagawa S, Ishikawa R, Ibuki E, Inoue K, Go T, Yokomise H, Ishii T, Kadowaki N, Haba R (2019). Limited resection is associated with a higher risk of locoregional recurrence than lobectomy in stage I lung adenocarcinoma with tumor spread through air spaces. Am J Surg Pathol.

[24] Eguchi T, Kameda K, Lu S, Bott MJ, Tan KS, Montecalvo J, Chang JC, Rekhtman N, Jones DR, Travis WD, Adusumilli PS (2019). Lobectomy is associated with better outcomes than sublobar resection in spread through air spaces (STAS)-positive T1 lung adenocarcinoma: A Propensity score-matched analysis. J Thorac Oncol.

[25] Warth A, Muley T, Kossakowski CA, Goeppert B, Schirmacher P, Dienemann H, Weichert W (2015). Prognostic impact of intra-alveolar tumor spread in pulmonary adenocarcinoma. Am J Surg Pathol.

[26] Lee JS, Kim EK, Kim M, Shim HS (2018). Genetic and clinicopathologic characteristics of lung adenocarcinoma with tumor spread through air spaces. Lung Cancer.

[27] Kadota K, Yeh YC, Sima CS, Rusch VW, Moreira AL, Adusumilli PS, Travis WD (2014). The cribriform pattern identifies a subset of acinar predominant tumors with poor prognosis in patients with stage I lung adenocarcinoma: a conceptual proposal to classify cribriform predominant tumors as a distinct histologic subtype. Mod Pathol.

[28] Kadota K, Kushida Y, Kagawa S, Ishikawa R, Ibuki E, Inoue K, Go T, Yokomise H, Ishii T, Kadowaki N, Haba R (2019). Cribriform subtype is an independent predictor of recurrence and survival after adjustment for the eighth edition of TNM staging system in patients with resected lung adenocarcinoma. J Thorac Oncol.

[29] Yanagawa N, Shiono S, Abiko M, Katahira M, Osakabe M, Ogata SY (2016). The clinical impact of solid and micropapillary patterns in resected lung adenocarcinoma. J Thorac Oncol.

[30] Cha MJ, Lee HY, Lee KS, Jeong JY, Han J, Shim YM, Hwang HS (2014). Micropapillary and solid subtypes of invasive lung adenocarcinoma: clinical predictors of histopathology and outcome. J Thorac Cardiovasc Surg.

[31] Hung JJ, Yeh YC, Jeng WJ, Wu KJ, Huang BS, Wu YC, Chou TY, Hsu WH (2014). Predictive value of the international association for the study of lung cancer/American Thoracic Society/European Respiratory Society classification of lung adenocarcinoma in tumor recurrence and patient survival. J Clin Oncol.

[32] Zombori T, Nyári T, Tiszlavicz L, Pálföldi R, Csada E, Géczi T, Ottlakán A, Pécsy B, Cserni G, Furák J (2018). The more the micropapillary pattern in stage I lung adenocarcinoma, the worse the prognosis-a retrospective study on digitalized slides. Virchows Arch.

[33] Medina MA, Onken AM, de Margerie-Mellon C, Heidinger BH, Chen Y, Bankier AA, VanderLaan PA (2020). Preoperative bronchial cytology for the assessment of tumor spread through air spaces in lung adenocarcinoma resection specimens. Cancer Cytopathol.

[34] Yeh YC, Nitadori J, Kadota K, Yoshizawa A, Rekhtman N, Moreira AL, Sima CS, Rusch VW, Adusumilli PS, Travis WD (2015). Using frozen section to identify histological patterns in stage I lung adenocarcinoma of ≤ 3 cm: accuracy and interobserver agreement. Histopathology.

[35] Morimoto J, Nakajima T, Suzuki H, Nagato K, Iwata T, Yoshida S, Fukuyo M, Ota S, Nakatani Y, Yoshino I (2016). Impact of free tumor clusters on prognosis after resection of pulmonary adenocarcinoma. J Thorac Cardiovasc Surg.

[36] Postmus PE, Kerr KM, Oudkerk M, Senan S, Waller DA, Vansteenkiste J, Escriu C, Peters S, ESMO Guidelines Committee (2017). Early and locally advanced non-small-cell lung cancer (NSCLC): ESMO Clinical practice guidelines for diagnosis, treatment and follow-up. Ann Oncol.

[37] http://ftsz.pte.hu/docs/protokollok/TUDOHorgorak.pdf. Accessed 30 Apr 2020

[38] Tsubokawa N, Mimae T, Miyata Y, Sasada S, Yoshiya T, Kushitani K, Takeshima Y, Murakami S, Yokose T, Ito H, Nakayama H, Okada M (2016). Prognostic significance of vascular invasion in intermediate-grade subtype of lung adenocarcinoma. Jpn J Clin Oncol.

