# Supplementary material for: Architectural Grade Combined With Spread Through Air Spaces (STAS) Predicts Recurrence and is Suitable for Stratifying Patients Who Might Be Eligible for Lung Sparing Surgery for Stage I Adenocarcinomas
Source: Pathol Oncol Res. 2020 Jun 20;26(4):2451–8. doi: 10.1007/s12253-020-00855-7 (PMC7471099; doi:10.1007/s12253-020-00855-7)
Supplement: Supplementary file 1 — (DOCX 12 kb) [file 12253_2020_855_MOESM1_ESM.docx]

*Supplementary table 1.* Multivariate Cox analysis of prognostic systems in lung adenocarcinoma (ARG-STAS: architectural grade combined with STAS, HR: hazard ratio, OS: overall survival, 95%CI: 95% confidence interval, DFS: disease-free survival)

|  | **p_OS_** | **HR_OS_** | **95%CI** | **p_DFS_** | **HR_DFS_** | **95%CI** |
| --- | --- | --- | --- | --- | --- | --- |
| **ARG-STAS** | **0.012** | **2.98** | **1.31-6.92** | **0.009** | **2.29** | **1.21-4.04** |
| **Architectural grade** | 0.702 | 1.25 | 0.39-4.02 | 0.316 | 1.51 | 0.67-3.38 |
| **Sica-grade** | 0.877 | 1.06 | 0.51-2.22 | 0.675 | 1.14 | 0.61-2.11 |
| **Kadota-grade** | 0.747 | 1.19 | 0.42-3.36 | 0.554 | 0.81 | 0.39-1.65 |
